# Perception of allergy in cinematography

**DOI:** 10.1186/1939-4551-8-S1-A1

**Published:** 2015-04-08

**Authors:** Georgios Mavroleon, Georgios Koutalonis

**Affiliations:** 1 Private clinic, Sfakion 10-12, 73134, Chania, Crete, Greece; 2 Private clinic, Tzanakaki 21, 73100, Chania, Crete, Greece

## Background

Allergic symptoms are presented in everyday life. Cinematography, as a form of art describing life, has many incidents regarding allergy and even mechanisms involved.

## Methods

Collect movies and TV series for the last 10 years, editing the few second parts, commenting on rightness or false of the screen play.

## Results

There are many referrals in allergic symptoms and patients, but not all the presentations are correct and this could lead to wrong perception in public of what allergy really is and how specialists could help.

## Conclusions

There is an increased interest in the presentation of allergic patients in movies and it is our duty to get involved and set the records staight.

